# A scoping review of military veterans involved in the criminal justice system and their health and healthcare

**DOI:** 10.1186/s40352-019-0086-9

**Published:** 2019-04-08

**Authors:** Andrea K. Finlay, Mandy D. Owens, Emmeline Taylor, Amia Nash, Nicole Capdarest-Arest, Joel Rosenthal, Jessica Blue-Howells, Sean Clark, Christine Timko

**Affiliations:** 1 Center for Innovation to Implementation, VA Palo Alto Health Care System, 795 Willow Road, Menlo Park, CA 94025 USA; 2 Department of Veterans Affairs, National Center on Homelessness Among Veterans, 795 Willow Road, Menlo Park, CA 94025 USA; 3 Department of Veterans Affairs Health Care System, Center of Innovation for Veteran-Centered and Value-Driven Care, 1660 S. Columbian Way, Seattle, WA 98108 USA; 4 Department of Health Services, University of Washington, 1959 NE Pacific St, Magnuson Health Sciences Center, Room H-680, Box 357660, Seattle, WA 98195-7660 USA; 5 Blaisdell Medical Library, University of California, Davis, 4610 X St, Sacramento, CA 95817 USA; 6 Veterans Justice Programs, Department of Veterans Affairs, 795 Willow Road, Menlo Park, CA 94025 USA; 7 Veterans Justice Programs, Department of Veterans Affairs, 11301 Wilshire Blvd, Los Angeles, CA 90073 USA; 8 Veterans Justice Programs, Department of Veterans Affairs, 2250 Leestown Road, Lexington, KY 40511 USA; 9 Department of Psychiatry and Behavioral Sciences, Stanford University School of Medicine, 401 Quarry Road, Stanford, CA 94305-5717 USA

**Keywords:** Military, Veterans, Criminal justice, Healthcare

## Abstract

**Background:**

In the criminal justice system, special populations, such as older adults or patients with infectious diseases, have been identified as particularly vulnerable to poor health outcomes. Military veterans involved in the criminal justice system are also a vulnerable population warranting attention because of their unique healthcare needs. This review aims to provide an overview of existing literature on justice-involved veterans’ health and healthcare to identify research gaps and inform policy and practice.

**Methods:**

A systematic search was conducted to identify research articles related to justice-involved veterans’ health and healthcare that were published prior to December 2017. Study characteristics including healthcare category, study design, sample size, and funding source were extracted and summarized with the aim of providing an overview of extant literature.

**Results:**

The search strategy initially identified 1830 unique abstracts with 1387 abstracts then excluded. Full-text review of 443 articles was conducted with 252 excluded. There were 191 articles included, most related to veterans’ mental health (130/191, 68%) or homelessness (24/191, 13%). Most studies used an observational design (173/191, 91%).

**Conclusions:**

Knowledge gaps identified from the review provide guidance on future areas of research. Studies on different sociodemographic groups, medical conditions, and the management of multiple conditions and psychosocial challenges are needed. Developing and testing interventions, especially randomized trials, to address justice-involved veterans care needs will help to improve their health and healthcare. Finally, an integrated conceptual framework that draws from diverse disciplines, such as criminology, health services, psychology, and implementation science is needed to inform research, policy and practice focused on justice-involved veterans.

**Electronic supplementary material:**

The online version of this article (10.1186/s40352-019-0086-9) contains supplementary material, which is available to authorized users.

## Public significance statement

Of 191 research articles published on the health and healthcare of veterans involved in the criminal justice system, the majority examined veterans’ mental health. Studies are needed that address challenges faced by different sociodemographic groups of veterans who are in the justice system and interventions that help them manage multiple mental health, substance use disorder, and medical conditions.

## Special populations in the criminal justice system

In the criminal justice system, vulnerable populations, such as women (Binswanger et al., 2010; Timko et al., 2019), older adults (Skarupski, Gross, Schrack, Deal, & Eber, 2018), and people with HIV or hepatitis C (Spaulding, Anderson, Khan, Taborda-Vidarte, & Phillips, 2017), are at risk of poor health outcomes. Although these groups have been the focus of numerous research studies, another vulnerable group – military veterans in the criminal justice system – only recently gained attention. Justice-involved veterans, which are military veterans detained by or under the supervision of the criminal justice system (e.g., incarcerated in jail or prison, supervised by probation or parole), are a special population who comprise approximately 8% of the incarcerated population in the U.S. (Bronson, Carson, Noonan, & Berzofsky, 2015). There are an estimated 181,500 veterans incarcerated in prisons and jails.

## Background on justice-involved veterans

Justice-involved veterans have extensive medical, mental health and substance use disorder treatment needs. Among veterans age 55 and older who were exiting prison, 50% had hypertension, 20% had diabetes, and 16% had hepatitis (Williams et al., 2010). In 2007, the Veterans Health Adminstration (VHA) implemented the Veterans Justice Programs, which are designed to connect justice-involved veterans with a broad range of VHA and community healthcare and related services. More than half of veterans seen by these programs are diagnosed with mental health or substance use disorders after entering VHA treatment (Finlay et al., 2016, 2017). The mortality risk among veterans exiting prison is similar to that of non-veterans exiting prison, 12 times higher than that of the general population, with overdose as the leading cause of death (Wortzel, Blatchford, Conner, Adler, & Binswanger, 2012).

In addition to healthcare treatment needs, justice-involved veterans face numerous psychosocial problems. Among veterans in prison, 30% have a history of homelessness (Tsai, Rosenheck, Kasprow, & McGuire, 2014). Housing can be difficult to find after incarceration, especially for veterans with registered sex offenses (U.S. Department of Veterans Affairs, 2015). Finding and maintaining employment can also be difficult. Challenges include legal restrictions on employment, stigma and criminal background checks, and competing needs and health conditions (McDonough, Blodgett, Midboe, & Blonigen, 2015). Finally, an estimated 10% of incarcerated veterans are not eligible for VHA services due to dishonorable/bad conduct or other discharge records from military service, and an additional 13% may not be eligible due to other than honorable discharge status (Bronson et al., 2015). These veterans may face the same challenges to finding healthcare as other justice-involved populations, including lack of insurance, limited community treatment options, or other competing challenges, such as finding and maintaining housing and employment (Mallik-Kane & Visher, 2008).

## Importance of examining justice-involved veterans

Examining the health and healthcare of justice-involved veterans is important for at least three reasons. First, veterans may have different healthcare treatment needs than civilians involved in the justice system because of combat or other traumatic events they experienced while in the military (Backhaus, Gholizadeh, Godfrey, Pittman, & Afari, 2016), which may have increased their risk for involvement in the criminal justice system. Second, the Veterans Health Administration (VHA), and society at large, has an obligation to care for veterans, including justice-involved veterans. Third, communities will be safer and save resources when the care needs of justice-involved veterans are addressed. Understanding the health and healthcare needs of incarcerated and other justice-involved veterans will allow the VHA and other settings in which veterans seek health care to design programming that will be responsive to the treatment priorities of these veterans.

## Differences between veterans and non-veterans

Prior research suggests some connection between military service and criminal justice involvement, which may be explained by profiles of those who volunteer for military service, traumatic experiences during military service, and medical, mental health, or substance use disorder conditions related to military service. People who volunteer for the military have higher odds of becoming incarcerated than people who do not join the military (Culp, Youstin, Englander, & Lynch, 2013). Pre-existing differences in people who join the military may also explain how type of crime committed varies by military status. Compared to non-veterans, a higher percentage of veterans were incarcerated in US prisons and jails for sexual offenses, but a lower percentage were incarcerated for property and drug offenses (Bronson et al., 2015). In Arizona, a higher percentage (30%) of veterans were arrested for a violent offense compared to non-veterans (20%) (White, Mulvey, Fox, & Choate, 2012).

Traumatic experiences and post-traumatic stress disorder (PTSD) have been linked with criminal justice involvement (Backhaus et al., 2016; Donley et al., 2012; Edalati & Nicholls, 2017; MacManus et al., 2013) and may explain the link between military service and criminal behaviors. People who select into the military may come from a background where they experienced more trauma. For example, compared to non-veterans, veterans experienced more adverse events in childhood (Katon et al., 2015). Among veterans in jail, 87% had experienced a lifetime traumatic event and 39% screened positive for PTSD (Saxon et al., 2001). In addition, exposure to combat or other traumatic situations may occur during military service. Among veterans in jail, 58% of men and 38% of women had served in a combat zone (Stainbrook, Hartwell, & James, 2016). Exposure to more traumatic events during military service and PTSD were linked with a higher risk of violent offending among veterans in the United Kingdom (MacManus et al., 2013). PTSD symptoms have been linked with interpersonal violence (Hoyt, Wray, & Rielage, 2014) and other criminal justice involvement among veterans (Brown, 2011) and civilians (Donley et al., 2012).

Health conditions related to military service may also be linked to criminal justice involvement. Traumatic brain injury, a signature injury of recent military conflicts (Snell & Halter, 2010), is associated with criminal behaviors, such as violent offending (Williams et al., 2018). Substance use disorders have been linked with recidivism among justice-involved veterans in the US (Blonigen et al., 2016; Tsai, Finlay, Flatley, Kasprow, & Clark, 2018) and post-deployment alcohol use has been associated with violent offending among veterans in the United Kingdom (MacManus et al., 2013). Among US veterans who served in recent conflicts in Iraq or Afghanistan, combat-related PTSD was linked with a high risk of incarceration (Tsai, Rosenheck, Kasprow, & McGuire, 2013a).

## Health differences between criminal justice involved veterans and non-veterans

There are few health differences between justice-involved veterans and non-veterans. In a sample of older adults leaving jail, prevalence rates of medical, mental health and substance use disorders were similar between incarcerated veterans and non-veterans, except veterans had a higher prevalence of asthma and PTSD (Williams et al., 2010). A nationally representative sample of men incarcerated in prisons and jails indicated that veterans had higher prevalence of a history of PTSD and personality disorders than non-veterans, but did not differ on a history of major depressive disorder, bipolar disorder, schizophrenia, or anxiety disorder (Bronson et al., 2015). Veterans and non-veterans released from prison shared a similar risk of death in the weeks immediately following release, though this risk was attenuated for veterans with VA benefits (Wortzel et al., 2012).

## Prior reviews on justice-involved veterans

Although there are prior reviews studies on justice-involved veterans, a scoping review has not yet been conducted. Scoping reviews aim to give a comprehensive view of the extant literature and identify gaps in research to inform research agendas and policy and practice (Arskey & O'Malley, 2005; Tricco et al., 2016). Prior reviews examined justice-involved veterans from a social work perspective (Canada & Albright, 2014), the socio-cultural and psychological aspects of military service and justice involvement (Brown, Stanulis, Theis, Farnsworth, & Daniels, 2013), the link between criminal behavior and military experience from a legal perspective (Holbrook & Anderson, 2011), the legal and clinical implications of PTSD among combat veterans (Fine & Levin, 2008; Marciniak, 1986), arrest rates among Vietnam veterans (Beckerman & Fontana, 1989), and theoretical models explaining criminal justice involvement among veterans with a summary of existing justice-related programming for veterans (Stacer & Solinas-Saunders, 2015). However, these reviews were not systematic. A few systematic reviews on specific topics related to justice-involved veterans have been conducted, including studies examining suicide risk (Wortzel, Binswanger, Anderson, & Adler, 2009), recidivism risk (Blonigen et al., 2016), and the prevalence of mental health disorders (Blodgett et al., 2015). A protocol for a systematic review to examine risk of criminal justice involvement among veterans with mental health and substance use disorder conditions was published by Taylor, Parkes, Haw, and Jepson (2012).

## Aims

The aim of the current study was to conduct a scoping review of the literature related to the health and healthcare of military veterans involved in the criminal justice system.. Accordingly, the purposes of this study were to: (1) Summarize and synthesize the extent and characteristics of the existing literature across multiple fields, and (2) Identify research gaps to be addressed in future research efforts. The results of this study will help guide future research in this area and inform policy and practice. Additionally, this reviewed literature can serve as a resource for researchers, legal professionals, healthcare providers, and other professionals who work with justice-involved veterans.

## Methods

The population of interest was justice-involved veterans, both in the US and internationally. As our aim was to summarize and synthesize the existing literature, we included all comparators, interventions, settings, and outcomes, but we did not evaluate, aggregate, or present study findings. Ethical approval is not required as our study includes only published peer-reviewed manuscripts and reports.

### Data sources and searches

Following a modified version of the PRISMA guidelines (Liberati et al., 2009) and guidelines for scoping reviews (Peters et al., 2015), we used a variety of search mechanisms to find articles related to justice-involved veterans. We searched five databases: MEDLINE/PubMed, Scopus, Web of Science, CINAHL, and PsychINFO. Keywords included *veterans* or *former military*, and criminal justice-related terms such as *prison, jail, court,* or *probation* with no restrictions on dates searched (see the Additional file 1 for search algorithms and terms used). The initial search was implemented on June 2, 2017. We also created alerts in the selected search engines and added articles through the study period ending November 30, 2017. During the summer of 2017, we queried experts in the field with requests for articles from their personal files. Finally, we mined references from articles to identify any missing work.

### Study selection

We excluded studies that did not include justice-involved veterans, were not relevant to health or healthcare, or were limited to active duty military personnel. Consistent with previous studies (Danan et al., 2017), we excluded several article types. Case reports, law articles/briefs, and meeting abstracts were excluded because most did not contain sufficient study description or results. Editorials, letters, protocols, and literature or systematic reviews were excluded because they did not include original empirical results. We also excluded brief news articles that did not report original results and articles that were not in English or without a published English translation. We included non-peer-reviewed publications if they were publicly available in a published form (e.g., government reports) and dissertations if they were publicly available and exhibited scientifically rigorous methods, but unpublished papers that were neither government reports or dissertations were excluded.

Prior to abstract review, duplicates were removed. The lead author (AKF) reviewed all abstracts with a co-investigator (MDO or CT) providing a secondary review. Any differences in agreement were discussed and resolved. Rayyan was used to review abstracts (Ouzzani, Hammady, Fedorowicz, & Elmagarmid, 2016). Full-text articles were obtained for the selected abstracts, and each article was independently reviewed by an investigator or research assistant. The lead author independently reviewed a 10% random sample of full-text articles that were reviewed by another investigator or research assistant and reviewed any additional articles when asked by the first reviewer. Any studies that raised questions were discussed among the research team to reach agreement.

### Data extraction

For studies that were selected for inclusion at the full-text stage, an investigator or research assistant extracted 14 study characteristics. We selected and defined these characteristics based on prior studies (Danan et al., 2017) and by conducting extraction with a subsample of articles and discussing potential characteristics to include. The final extracted characteristics were: (1) healthcare category, (2) study design, (3) sample size, (4) percentage of veterans, (5) number of justice-involved veterans, (6–8) reporting of gender, race and age, (9) research setting, (10) period of military service, (11) outcomes reported, (12) funding source, (13) country, and (14) period of data collection.

### Data synthesis and analysis

We summarized the selected studies across characteristics. Consistent with the aims of a scoping review, our results present an overview of the extant literature, but we do not examine individual studies. Thus, we did not assess for risk of bias nor did we analyze the strength of the evidence.

## Results

**Fig. 1:**
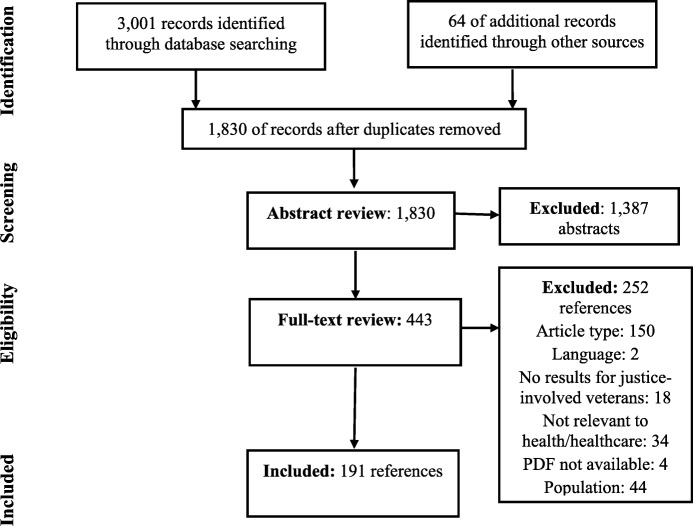
Adapted flow chart of record identification and screening process

In total, we reviewed 1830 abstracts and excluded 1387 abstracts (Fig. 1). We reviewed full-texts of 443 articles, of which 252 were excluded. All included studies are listed in Table 1 and summarized by healthcare category, sample size, study design, and funding source. The majority of studies were related to mental health (130/191, 68%) or homelessness (24/191, 13%). There were 49 studies (26%) published prior to 2000, 55 articles (29%) published from 2000 to 2012, and 86 articles (45%) published from 2013 to 2017. The majority (133/191, 70%) of articles drew from samples in VHA treatment settings or programs, but the remaining 30% were conducted in other settings where justice-involved veterans seek healthcare including jails, prisons, courts, and community treatment settings.

**Table 1:** Healthcare Categories and Topics of Reviewed Articles

Healthcare Topics Author Name (Year)	
Mental Health – 130 articles total	
Homelessness	
Copeland et al. (2009)	
Harpaz-Rotem, Rosenheck, and Desai (2006)	
Mental healthcare programming	
Neale and Rosenheck (2000)	
Multiple mental health, substance use disorder and/or medical conditions	
Benda et al. (2003a)	
Black et al. (2005)	
Boivin (1987)	
Bromley et al. (2017)	
Bronson et al. (2015)	
Brooke and Gau (2018)	
Brown and Jones (2015)	
Brown and Jones (2016)	
Cacciola et al. (1994)	
Erickson, Rosenheck, Trestman, Ford, and Desai (2008)	
Finlay et al. (2015)	
Finlay, Smelson, et al. (2016)	
Finlay et al. (2017)	
Gauthier (2017)	
Greenberg and Rosenheck (2009)	
Kimbrel et al. (2014)	
McLellan, Erdlen, Erdlen, and O'Brien (1981)	
Mohamed (2013)	
Mumola (2000)	
Nace, Meyers, O'Brien, Ream, and Mintz (1977)	
Noonan and Mumola (2007)	
Otis and Louks (2001)	
Pandiani, Ochs, and Pomerantz (2010)	
Pandiani, Rosenheck, and Banks (2003)	
Rosenheck, Banks, Pandiani, and Hoff (2000)	
Schaffer (2011)	
Schaffer (2014)	
Schaffer (2016b)	
Schuckit, Miller, and Hahlbohm (1975)	
Timko et al. (2017)	
Tsai and Goggin (2017)	
Tsai, Rosenheck, et al. (2013a)	
Tsai, Rosenheck, Kasprow, and McGuire (2013b)	
Weinstock and Nair (1984)	
White et al. (2012)	
Wilson and Walker (1990)	
Yesavage (1983)	
Other mental health topics	
Rosenheck, Frank, and Graber (1987)	
PTSD and/or trauma	
Backhaus et al. (2016)	
Bennett et al. (2018)	
Brown (2011)	
Cantrell (1999)	
Coker and Rosenheck (2014)	
Elbogen, Johnson, Newton, et al. (2012)	
Fontana and Rosenheck (2008)	
Harmless (1990)	
Hartwell et al. (2014)	
Heinz, Cohen, Holleran, Alvarez, and Bonn-Miller (2016)	
Johnson et al. (1996)	
Larson and Norman (2014)	
Saxon et al. (2001)	
Schry et al. (2015)	
Shaw, Churchill, Noyes Jr, and Loeffelholz (1987)	
Sherman, Fostick, Zohar, and Israeli Consortium (2014)	
Sigafoos (1994)	
Silverstein (1996)	
Stainbrook et al. (2016)	
Wilson and Zigelbaum (1983)	
Zeber, Noel, Pugh, Copeland, and Parchman (2010)	
Race/ethnicity	
Greenberg and Rosenheck (2012)	
Greenberg, Rosenheck, and Desai (2007)	
Substance use disorders	
Atkinson et al. (1993)	
Bale et al. (1980)	
Bale et al. (1984)	
Bray, O'Malley, Ashcroft, Adedeji, and Spriggs (2013)	
Cacciola, Alterman, Rutherford, McKay, and McLellan (1998)	
Calsyn, Roszell, and Chaney (1989)	
Comings, Muhleman, Ahn, Gysin, and Flanagan (1994)	
Cote (2013)	
Craig (1980)	
Davis et al. (2017)	
Davis et al. (2003)	
Decker, Peglow, and Samples (2014)	
Derkzen and Wardop (2015)	
Finlay et al. (2018)	
Finlay, Harris, et al. (2016)	
Groppenbacher, Batzer, and White (2003)	
Hser et al. (2006)	
Joe and Hudiburg (1978)	
Kasarabada, Anglin, Stark, and Paredes (2000)	
Khalsa, Kowalewski, Anglin, and Wang (1992)	
Laudet, Timko, and Hill (2014)	
McKay et al. (1998)	
McKay, Merikle, Mulvaney, Weiss, and Koppenhaver (2001)	
McLellan, Ball, Rosen, and O'Brien (1981)	
McLellan, Luborsky, O'Brien, Barr, and Evans (1986)	
McLellan, Luborsky, O'Brien, Woody, and Druley (1982)	
McQuaid et al. (2000)	
Moore et al. (2017)	
Richards et al. (1990)	
Rogalski (1987)	
Rothbard et al. (1999)	
Santos, Martinez, and Perez (1998)	
Schultz, Blonigen, Finlay, and Timko (2015)	
Siegal et al. (1995)	
Siegal, Li, and Rapp (2002)	
Snowden, Oh, Salas-Wright, Vaughn, and King (2017)	
Stack, Cortina, Samples, Zapata, and Arcand (2000)	
Wallace and Weeks (2004)	
Wang, Hieb, and Wildt (1976)	
Weaver, Trafton, Kimerling, Timko, and Moos (2013)	
Xu et al. (2016)	
Yager, Laufer, and Gallops (1984)	
Yates, Booth, Reed, Brown, and Masterson (1993)	
Suicide	
Ilgen et al. (2007)	
Veterans Treatment Courts	
Clark et al. (2014)	
Clifford et al. (2014)	
Gallagher et al. (2016)	
Johnson, Stolar, Wu, Coonan, and Graham (2015)	
Johnson, Graham, Sikes, Nelsen, and Stolar (2015)	
Johnson et al. (2017)	
Knudsen and Wingenfeld (2016)	
Slattery, Dugger, Lamb, and Williams (2013)	
Tsai, Flatley, et al. (2017)	
Violence	
Anderson et al. (2017)	
Elbogen, Johnson, Wagner, et al. (2012)	
Gerlock (2004)	
Hiley-Young, Blake, Abueg, Rozynko, and Gusman (1995)	
Hoyt et al. (2014)	
Lynch and Noel (2010)	
MacManus et al. (2013)	
Mays, Gordon, Kelly, and Forman (2006)	
Peralme (1995)	
Rohlfs (2010)	
Schaffer (2016a)	
Stacer and Solinas-Saunders (2015)	
Sullivan and Elbogen (2014)	
Homelessness – 24 articles total	
Death	
Montgomery et al. (2016)	
Multiple mental health, substance use disorders and/or medical conditions	
Cusack and Montgomery (2017)	
Douyon et al. (1998)	
Gabrielian et al. (2016)	
Seidner, Burling, Fisher, and Blair (1990)	
Stovall, Cloninger, and Appleby (1997)	
Tejani et al. (2014)	
Tsai, Kasprow, and Rosenheck (2013)	
Tsai et al. (2011)	
Tsai and Rosenheck (2013b)	
Tsai and Rosenheck (2016)	
Tsai, Rosenheck, and Kane (2014)	
Tsai, Rosenheck, Kasprow, and McGuire (2014)	
Wenzel et al. (1996)	
Wenzel et al. (1993)	
Williams et al. (2010)	
Other mental health topics	
Tsai and Rosenheck (2013a)	
PTSD and/or trauma	
Hamilton, Poza, and Washington (2011)	
Substance use disorders	
Benda, Rodell, and Rodell (2003b)	
Benda, Rodell, and Rodell (2003c)	
Westermeyer and Lee (2013)	
Winn et al. (2013)	
Violence	
Schaffer (2012)	
Vocational training	
Kashner et al. (2002)	
Access & Utilization – 14 articles total	
Barriers and facilitators of care	
Blonigen et al. (2018)	
Butt et al. (2005)	
Wainwright et al. (2017)	
Death	
Wortzel et al. (2012)	
Health care utilization	
DeViva (2014)	
McGuire (2008)	
McGuire et al. (2003)	
Petrovich et al. (2014)	
Trojano et al. (2017)	
Tsai, Middleton, et al. (2017)	
Wang et al. (2015)	
Veterans Treatment Courts	
Shannon et al. (2017)	
Tsai et al. (2018)	
Violence	
Gerlock (1999)	
Medical – 10 articles total	
Brain injury	
(Virkkunen et al., 1977)	
Death	
LePage, Bradshaw, et al. (2016)	
Luallen and Corry (2017)	
Hypertension	
Howell et al. (2016)	
Infectious diseases	
Briggs et al. (2001)	
Cheung et al. (2002)	
Currie (2009)	
Dominitz et al. (2005)	
Mishra et al. (2003)	
Other medical topics	
Mazur (1995)	
Psychosocial – 10 articles	
Homelessness	
Elbogen, Sullivan, Wolfe, Wagner, and Beckham (2013)	
Multiple mental health, substance use disorders and/or medical conditions	
Reinemann (1947)	
PTSD and/or trauma	
Wilson, Draine, Hadley, Metraux, and Evans (2011)	
Screening in primary care	
Bikson et al. (2009)	
Cook et al. (1996)	
Vocational training	
LePage et al. (2014)	
LePage, Lewis, et al. (2016)	
LePage et al. (2017)	
LePage et al. (2013)	
LePage et al. (2011)	
Healthcare Organization & Delivery – 2 articles total	
Mental healthcare programming	
Stainbrook, Penney, and Elwyn (2015)	
Peer support	
Clark et al. (2016)	
Long-term Care/Aging – 1 article total	
Mental healthcare programming	
Kopera-Frye et al. (2013)	

### Healthcare category

#### Mental health conditions

Substance use disorders were the most common conditions examined in studies, including studies focused solely on veterans with alcohol use disorder (Finlay et al., 2018; McQuaid et al., 2000; Moore, Fuehrlein, & Rosenheck, 2017; Richards, Goldberg, Anderson, & Rodin, 1990), opioid use disorder (Craig, 1980; Finlay et al., 2016; Rothbard et al., 1999), or co-occurring substance use and other mental health diagnoses (Mohamed, 2013; Timko, Finlay, Schultz, & Blonigen, 2017; Wenzel et al., 1996). Some studies investigated the prevalence of multiple mental health and substance use disorder conditions, reporting on these conditions and healthcare utilization among justice-involved veterans (e.g., Finlay et al., 2017; Finlay, Smelson, et al., 2016).

Several articles focused on conditions and experiences related to military service such as PTSD and trauma (Backhaus et al., 2016; Bennett, Morris, Sexton, Bonar, & Chermack, 2018; Elbogen et al., 2012; Saxon et al., 2001; Sigafoos, 1994). A number of observational studies addressed violence (Elbogen et al., 2012; Hoyt et al., 2014; MacManus et al., 2013) and Veterans Treatment Courts (Clark, Blue-Howells, & McGuire, 2014; Knudsen & Wingenfeld, 2016; Tsai, Flatley, Kasprow, Clark, & Finlay, 2017). Only one study we identified examined suicide as the primary outcome (Ilgen, Harris, Moos, & Tiet, 2007), though another study addressed suicide along with other factors (Kimbrel et al., 2014).

#### Homelessness

Studies were coded as fitting the healthcare category of Homelessness when the samples examined were homeless veterans (i.e., veterans who were homeless prior to treatment or who were receiving homeless services) or the study’s primary focus was to examine homelessness. The majority of participants in these studies were currently or previously justice-involved and/or their criminal justice involvement was a primary or secondary factor in the study. Often these veterans were recruited from VHA clinical settings, such as addiction treatment programs (Benda, Rodell, & Rodell, 2003a) or mental health inpatient care (e.g., Douyon et al., 1998), or were from VHA homeless programs (e.g., Cusack & Montgomery, 2017; Gabrielian et al., 2016; Tsai, O'Connell, Kasprow, & Rosenheck, 2011), although four studies were conducted in non-VHA settings (e.g., Montgomery, Szymkowiak, Marcus, Howard, & Culhane, 2016; Williams et al., 2010). Mental health and substance use disorders were the most commonly addressed issues among these studies, though some studies also examined medical conditions.

#### Access & utilization

Of the 14 articles that examined access and utilization, half reported on healthcare service use, such as treatment utilization among veterans with PTSD who recently returned from military service in Iraq or Afghanistan (DeViva, 2014), differences in treatment use among veterans who received VHA outreach services while in jail compared to veterans who received VHA outreach services in settings to address homelessness (McGuire, Rosenheck, & Kasprow, 2003), and health services utilization among veterans who received medical-legal partnership services (Tsai et al., 2017). Five of the studies occurred in non-VHA settings, including prison (Wainwright, McDonnell, Lennox, Shaw, & Senior, 2017; Wortzel et al., 2012), an emergency shelter (Petrovich, Pollio, & North, 2014), and courts (Shannon et al., 2017; Trojano, Christopher, Pinals, Harnish, & Smelson, 2017). Three articles described barriers to and facilitators of healthcare for justice-involved veterans (Blonigen et al., 2018; Butt, Wagener, Shakil, & Ahmad, 2005; Wainwright et al., 2017).

#### Medical

Infectious diseases were the most commonly addressed medical conditions. Five studies were of veterans who had HIV or hepatitis C and incarceration was examined as a risk factor (Cheung, Hanson, Maganti, Keeffe, & Matsui, 2002; Currie, 2009; Dominitz et al., 2005; Mishra, Sninsky, Roswell, Fitzwilliam, & Hyams, 2003). Mortality while in prison (Luallen & Corry, 2017) and after exiting prison (LePage, Bradshaw, Cipher, Crawford, & Parish-Johnson, 2016) was examined. Other medical conditions examined included brain injury (Virkkunen, Nuutila, & Huusko, 1977), hypertension (Howell et al., 2016) and hormone levels related to antisocial behavior (Mazur, 1995).

#### Psychosocial

Vocational training was the most commonly studied psychosocial factor in relation to the health of justice-involved veterans. The majority of studies were randomized controlled trials comparing vocational training to usual care among justice-involved veterans in VHA settings (LePage et al., 2016, 2017; LePage, Lewis, Washington, Davis, & Glasgow, 2013; LePage, Ottomanelli, Barnett, & Njoh, 2014; LePage, Washington, Lewis, Johnson, & Garcia-Rea, 2011). Screening for psychosocial issues in primary care was addressed by two studies (Bikson, McGuire, Blue-Howells, & Seldin-Sommer, 2009; Cook, Freedman, Freedman, Arick, & Miller, 1996).

### Sample size

Study sample sizes were somewhat evenly distributed with 21% (41/191) of studies with fewer than 100 participants, 40% (77/191) of studies with 100–1000 participants, and 38% (73/191) of studies with over 1000 participants. Of studies with fewer than 100 participants, the majority took place at a single VHA site (13/41; 32%) or at a single court (or multiple courts (9/41; 22%) with additional studies conducted in jail or prison settings or in multiple settings. Of studies with 100–1000 participants, 61% (47/77) were conducted at a single VHA site and 15% (11/77) were conducted in non-VHA settings. Of studies with over 1000 participants, 36% (26/73) used VHA administrative/clinical databases, 22% (16/73) used multiple data sources (e.g., prison release records and VHA administrative databases), and 18% (13/73) collected data from multiple VHA sites. The remaining studies were conducted in prison or jail settings, drew from multiple data sources, or were surveys of veterans.

### Research settings

Studies in VHA settings that used administrative databases identified veterans who participated in a VHA justice program (e.g., Finlay et al., 2015; Tsai et al., 2018) or who reported a criminal justice history (Gabrielian et al., 2016; Tejani, Rosenheck, Tsai, Kasprow, & McGuire, 2014). Court mandates for treatment were not recorded in these databases. Single VHA site studies used criminal justice data gathered through randomized controlled trials (Anderson et al., 2017; Bennett et al., 2018), longitudinal treatment surveys (Atkinson, Tolson, & Turner, 1993; Timko et al., 2017), and one-time questionnaires (Backhaus et al., 2016; Briggs et al., 2001). Multiple VHA site studies included qualitative interviews (Blonigen et al., 2018), longitudinal assessments (Tsai, Middleton, et al., 2017), and administrative evaluation data (Coker & Rosenheck, 2014).

### Study design

The majority of studies identified for this review used an observational design (173/191; 91%). Ten studies (5%) used a randomized clinical trial design with an additional two studies (1%) conducting secondary analyses of a randomized clinical trial. Six studies (3%) used qualitative interviewing and focus group methods.

### Funding source

The majority of studies either did not report a funding source (97/191, 51%) or were unfunded (5/191, 3%). When reported, the most common funding sources were VHA funding (53/191, 28%), National Institutes of Health funding (31/191, 16%), and other government funding (21/191, 11%) including the Department of Defense and the Substance Abuse and Mental Health Services Administration. The remaining funding support was from universities (6/191, 3%) and foundations (6/191, 3%).

## Discussion

This scoping review summarizes the research literature on justice-involved veterans and their health and healthcare. The majority of studies focused on mental health conditions, and over 90% used an observational research design. Few studies examined medical conditions, psychosocial factors, healthcare delivery and organization, or long-term care and aging in this vulnerable population. Randomized clinical trials aimed at improving health outcomes, rather than simply observing and documenting outcomes, were rare. Half of studies did not report a funding source or were unfunded, 28% of studies were funded by the VHA, and 27% were supported by other government funding.

### PTSD, military service, and criminal justice involvement

Mental health conditions, particularly PTSD and substance use disorders, were the foci of most articles published in the justice-involved veterans’ scientific literature. PTSD was consistently linked to more legal problems among US veterans (Backhaus et al., 2016; Black et al., 2005; Saxon et al., 2001). PTSD and combat exposure were significantly associated with violent offending among military veterans in the UK (MacManus et al., 2013). Similarly, among US veterans, PTSD and “anger hyperarousal symptoms” (derived from the Davidson Trauma Scale question that asked in the past week “Have you been irritable or had outbursts of anger?”) were found to predict family violence across a one-year study period (Sullivan & Elbogen, 2014). Among US veterans who served in Iraq or Afghanistan, military sexual trauma was linked with higher predicted probability of legal problems (Backhaus et al., 2016). Prosecutors offered more diversion programs to veterans with PTSD and thought they were less criminally culpable than veterans without PTSD (Wilson, Brodsky, Neal, & Cramer, 2011).

Combat exposure – and related PTSD from such experiences – was examined to explain the link between military experience and criminal justice involvement, though results were mixed. Combat experience has been associated with lower odds of non-violent offending (Bennett et al., 2018), and serving in wartime has been linked with lower odds of incarceration (Culp et al., 2013). However, greater combat exposure has also been associated with higher odds of unlawful behavior, including “having been arrested”, “being on probation or parole”, or “driving a car or other vehicle after having too much to drink” (Larson & Norman, 2014). While neither causal nor conclusive, this body of research on PTSD and combat exposure suggests that systems serving veterans should increase access to evidence-based trauma treatment for justice-involved veterans and develop prevention programs to attenuate their risk for violence and justice involvement.

Other aspects of military service were examined in relation to criminal justice involvement and were similarly inconclusive. Compared to enlisted soldiers, officers had lower odds of being incarcerated (Black et al., 2005) or of violent offending (MacManus et al., 2013). In most studies, period of service was either not specified or included veterans from multiple periods of service without examining differences by service era. One exception was a study that compared veterans from Iraq/Afghanistan, Gulf War, and Vietnam eras: Veterans who served during the Iraq/Afghanistan era had a lower rate of incarceration than veterans from the other eras of service (Fontana & Rosenheck, 2008). Branch of service was mentioned in a few studies. For example, a higher percentage of veterans incarcerated in jail served in the Army or Marines compared to veterans who were not incarcerated (Greenberg & Rosenheck, 2009). Length of service was examined with longer military service associated with fewer lifetime arrests among veterans incarcerated in prison (Brooke & Gau, 2018). However, examination of aspects of military service and links with criminal justice involvement were rare.

Although more research is needed to explore the link between military service and criminal justice involvement, results will have implications for the Department of Defense in their treatment of active duty personnel. For example, if combat trauma is determined to be a mechanism that causes later criminal justice involvement, designing post-deployment treatment programs that comprehensively address PTSD and trauma experienced while personnel are still serving in the military will be an important practice change. The VHA could use the reviewed studies to estimate the number of veterans who may become justice-involved and allocate treatment services to help reduce criminal behavior.

### Knowledge gaps and informing policy, practice, and research

The scoping review uncovered numerous gaps in the literature on the health and healthcare of justice-involved veterans. These gaps include studies of different sociodemographic groups, and research on veterans’ medical conditions and the impact of managing multiple medical, mental health, and substance use disorder conditions. Gaps were also apparent for studies of interventions to improve the health and healthcare of justice-involved veterans, especially studies using randomized trials. Differences in health and healthcare by type of criminal justice involvement were understudied. Conceptual models were rarely used to guide studies’ analyses or interpretation of results, and there was little consistency across studies that used conceptual models. The identified gaps provide guidance on areas for future research.

#### Medical conditions

Needed are studies focused on medical conditions, especially conditions such as traumatic brain injury, which may disproportionately affect veterans and be related to their justice involvement (To et al., 2015). Research on traumatic brain injury in veterans will also be relevant to both veterans and non-veterans with justice involvement as this condition is prevalent among justice-involved populations (Durand et al., 2017). Although hypertension is the most common medical condition among veterans served at the VHA (Frayne et al., 2014), only one study touched on this topic (Howell et al., 2016). Other chronic medical conditions, such as diabetes, were unaddressed in the studies we reviewed and need attention in future research. Studies on suicide and suicide risk will inform programming by the VHA Office of Suicide Prevention. Other important topics that need research include mortality, and the impact of civil legal issues on criminal issues and health. For example, studies on medical-legal partnerships (Tsai, Middleton, et al., 2017) may shed light on the types of civil legal issues that are most effectively addressed among veterans, allowing legal providers to be strategic with their time and resources.

#### Management of multiple conditions

Even though chronic mental health or addiction conditions, including depression and alcohol use disorder, were examined in a number of studies, the long-term management of these conditions in clinical practice among justice-involved veterans is an area of untapped investigation. A subset of studies examined multiple medical, mental health, and substance use disorder conditions, however, most lacked in-depth analysis on the topic, only reporting the prevalence of such conditions and health services utilization. Some studies examined the interaction of these conditions. However, given that 35–58% of justice-involved veterans served by VHA outreach programs have co-occurring mental health and substance use disorders (Finlay et al., 2017; Finlay, Smelson, et al., 2016) and many have medical conditions (Brown & Jones, 2015), more studies are needed that examine the cumulative effect of managing multiple conditions to inform clinical practice and policy. Studies that investigate how cycling in and out of incarceration impacts management of multiple conditions are also important. Furthermore, many justice-involved veterans who have mental health and addiction conditions struggle with homelessness and unemployment. Although the VHA and community programs provide comprehensive housing and employment training services to some justice-involved veterans, the impact of these programs, especially for veterans with multiple chronic mental health and addiction conditions, is unknown. Efforts to identify and evaluate approaches to meeting housing and employment needs across the spectrum of justice-involved veterans will be critical to improving the health of this population by means of improved clinical practice and evolving policy decisions.

#### Sociodemographic differences

The scoping review highlights that we need to know more about sociodemographic groups within the justice-involved veteran population, such as women, people of color, rural veterans, veterans with disabilities, and veterans from different periods of service and service branches. Only a few studies examined women veterans separately from men (Finlay et al., 2015; Stainbrook et al., 2016) and only one study was of transgender compared to non-transgender veterans (Brown & Jones, 2015). To inform clinical practice and policy, research is needed to examine the extent to which these underrepresented veterans differ from white male veterans living in urban areas, who have predominated in most justice-related studies, what unique programmatic needs they may have, and the effectiveness of tailored intervention programs.

#### Intervention studies

Along with studies on sociodemographic groups, intervention studies focused on addressing the unique and additional treatment needs of justice-involved veterans and preventing or reducing their criminal justice involvement are needed. There is a robust literature examining the link between criminal justice involvement and mental health and addiction issues (e.g., Baillargeon, Binswanger, Penn, Williams, & Murray, 2009; Binswanger et al., 2012), and the effectiveness of interventions to improve outcomes among the general population of justice-involved individuals (e.g., Cusack, Morrissey, Cuddeback, Prins, & Williams, 2010; Kinlock et al., 2007). Borrowing from this literature to inform policy and practice with veterans, as well as developing this body of research among veterans will help move the field of justice-involved veterans research forward. Expanding the study designs used to include more randomized controlled trials, qualitative studies such as interviews or focus groups, and more rigorous observational studies that allow for propensity score analysis and other sophisticated statistical tests are needed.

Although 30% of studies focused on veterans in non-VHA settings, information on the quality of health and healthcare of justice-involved veterans in non-VHA treatment setting is lacking, as is best practices for how to coordinate between VHA and non-VHA treatment settings. The lack of studies in non-VHA settings may be partially because most healthcare provided to veterans occurred at VHA facilities. However, in 2014, Congress enacted the Veterans Access, Choice, and Accountability Act, known as the Veterans Choice Program, which enabled the VHA to substantially expand the purchase of community care for veterans. Primary care and mental health care, including substance use disorder care, were among the top five types of community care used by veterans (Vanneman et al., 2017). In 2018, the VA MISSION Act continued funding for the Veterans Choice Program and an additional 640,000 veterans are estimated to move into community care annually in the early years of the program (Rieselbach, Epperly, Nycz, & Shin, 2019). Understanding what impact purchased care has on justice-involved veterans and coordination between VHA and non-VHA treatment is important to ensuring they are receiving high quality care (Liu et al., 2010).

#### Type of criminal justice involvement

Distinctions in the health and healthcare among veterans involved in different aspects of the criminal justice system are difficult to draw because the majority of articles did not examine differences by criminal justice type. Most articles asked about current (Backhaus et al., 2016; Cook et al., 1996) or past criminal justice involvement, such as lifetime legal problems measured by the Addiction Severity Index (Anderson et al., 2017; Benda et al., 2003a; Bennett et al., 2018; Cacciola, Rutherford, Alterman, & Snider, 1994), and results were not reported by type of criminal justice involvement. Studies also examined veterans in jail diversion programs (Clark, Barrett, Frei, & Christy, 2016; Hartwell et al., 2014), courts (Clifford, Fischer, & Pelletier, 2014; Gallagher, Nordberg, & Gallagher, 2016; Hoyt et al., 2014), or jails (Davis, Baer, Saxon, & Kivlahan, 2003; Greenberg & Rosenheck, 2009; Saxon et al., 2001), but these studies often lacked comparison groups.

There were several articles that studied veterans incarcerated in prison, but samples were limited to US state prisons only with no comparisons with US federal prisons (Boivin, 1987; Brooke & Gau, 2018; Luallen & Corry, 2017; Stacer & Solinas-Saunders, 2015; Tsai & Goggin, 2017) or it was not stated where the incarceration occurred (Black et al., 2005). One exception was a study found that a higher percentage of jail incarcerated veterans had current indicators of mental health problems and more previous mental health problems than prison incarcerated veterans (Bronson et al., 2015). From the broader criminal justice literature, limited evidence suggests that individuals incarcerated in prisons have similar or greater medical needs (Maruschak, Berzofsky, & Unangst, 2015), but have fewer mental health needs compared to individuals in jails (Bronson & Berzofsky, 2017). Individuals in prison also may have greater access to healthcare than those in jails or under community supervision, including medical care (Maruschak et al., 2015) and substance use treatment (Taxman, Perdoni, & Harrison, 2007), which likely is related to being in a confined environment for longer sentences. However, given that the majority of justice-involved individuals are under community supervision (71%) (Kaeble, Glaze, Tsoutis, & Minton, 2016), future work should identify and better understand potential differences in health and healthcare by criminal justice status.

#### Conceptual models

Although the majority of studies in our scoping review lacked a conceptual model, a few studies drew from conceptual models across a variety of fields. One study grounded their research in criminology models, including the importation model and the functionalist model (Stacer & Solinas-Saunders, 2015). Conceptual models drawn from psychology included psychosocial rehabilitation (Elbogen, Johnson, Wagner, et al., 2012), ecological theory and cross-cultural approaches (Clifford et al., 2014), and a survivor mode coping model (Wilson & Zigelbaum, 1983). Two health services studies used the Gelberg-Andersen Behavioral Model for Vulnerable Populations (Gabrielian et al., 2016; Petrovich et al., 2014). Finally, the RE-AIM (Reach, Effectiveness, Adoption, Implementation, and Maintenance) model was used in an implementation science study (Blonigen et al., 2018). The field of justice-involved veterans draws from different disciplines with their own conceptual models, but the lack of a common framework is a notable gap. Convening an interdisciplinary research consortium to develop a unifying conceptual model will help integrate these disciplines and guide future research.

### Limitations of the scoping review

This scoping review was designed to provide a broad overview of the literature on the health and healthcare of justice-involved veterans and how these articles add to our general understanding of criminal justice involved populations. We did not provide an in-depth analysis of the topics covered in the reviewed studies, the quality of these studies, or an investigation of bias; thus, we were limited in the conclusions we could draw about the research we reviewed. We did not conduct a second review of all full-text articles; rather, a second review was conducted on only a subset of articles. Finally, we limited our search to healthcare databases. Additional articles relevant to our review may have been published in other fields, such as law journals, and not every relevant article may have been identified using our search strategy. Articles not available in English that may have been relevant were also excluded due to limitations on the availability of translation. However, the search strategy used likely identified most key studies available in English and the findings likely reflect the scope of healthcare issues related to justice-involved veterans currently in the literature. Many of the articles we excluded focused on legal aspects of veterans’ experiences in the criminal justice system, such as recidivism and legal rationales for considering PTSD when charging a veteran. Criminal justice outcomes were included in some of the studies in our scoping review, though we did not summarize those outcomes here. We instead focused our review on health and healthcare outcomes, but a more comprehensive review of the literature including health, law, and other related areas, such as sociology, may be needed to fully understand the experiences of justice-involved veterans.

## Conclusions

Identifying and organizing existing literature to inform current research, and strategically expanding into existing gaps, will help to generate a robust body of literature focused on the health and healthcare of justice-involved veterans. The current review identified gaps in the justice-involved veteran literature, which also may exist in the general literature on justice-involved individuals, and highlighted areas for future research. Accomplishment of research in the identified domains will help inform policy and practice to improve the health and healthcare of justice-involved veterans as well as treatment for other justice-involved populations who have similar experiences of trauma and mental health and addiction issues.

## Additional file

Additional file 1: Database search algorithms. (DOCX 16 kb)

## Abbreviations

PTSD: Post-traumatic stress disorder
VHA: Veterans Health Administration
