# Supplementary material for: A scoping review of military veterans involved in the criminal justice system and their health and healthcare
Source: Health Justice. 2019 Apr 8;7:6. doi: 10.1186/s40352-019-0086-9 (PMC6718001; doi:10.1186/s40352-019-0086-9)
Supplement: Supplementary file 1 — Database search algorithms. (DOCX 16 kb) [file 40352_2019_86_MOESM1_ESM.docx]

Additional file 1

Database search algorithms

a) MEDLINE/PubMed

(“veterans”[mesh] OR veteran*[tw] OR “former military”[tw]) AND (“criminals”[mesh] OR criminal*[tw] OR “prisoners”[mesh] OR prison*[tw] OR jail*[tw] OR imprison*[tw] OR incarcerat*[tw] OR offender*[tw] OR convict*[tw] OR inmate*[tw] OR parole*[tw] OR probation[tw] OR recidiv*[tw] OR court*[tw] OR justice[tw] OR legal[tw] OR diversion[tw] OR violation[tw] OR dui[tw])

b) Scopus

( TITLE ( veteran*  OR  “former military” )  AND  TITLE-ABS-KEY ( criminal*  OR  prison*  OR  jail*  OR  imprison*  OR  incarcerat*  OR  offender*  OR  convict*  OR  inmate*  OR  parole*  OR  probation  OR  recidiv*  OR  court*  OR  justice*  OR  legal  OR  diversion  OR  violation  OR  dui ) )

c) Web of Science

( TI=( veteran* OR “former military” ) AND TS=( criminal* OR prison* OR jail* OR imprison* OR incarcerat* OR offender* OR convict* OR inmate* OR parole* OR probation OR recidiv* OR court* OR justice* OR legal OR diversion OR violation OR dui ) )

d) CINAHL

(veteran* OR “former military”) AND (criminal* OR prison* OR jail* OR imprison* OR incarcerat* OR offender* OR convict* OR inmate* OR parole* OR probation OR recidiv* OR court* OR justice* OR legal OR diversion OR violation OR dui)

e) PsycINFO

((exp MILITARY VETERANS/ OR veteran*.m_titl.) AND  (exp Correctional Institutions/ or exp Prisoners/ or exp Incarceration/ or exp Prisons/ or (criminal* or prison* or jail* or imprison* or incarcerat* or offender* or convict* or inmate* or parole* or probation or recidiv* or court* or justice* or legal or diversion or violation or dui).mp.))
